# Glycerophosphocholine provision rescues *Candida albicans* growth and signaling phenotypes associated with phosphate limitation

**DOI:** 10.1128/msphere.00231-23

**Published:** 2023-10-16

**Authors:** William R. King, Maikel Acosta-Zaldívar, Wanjun Qi, Nicholas Cherico, Lauren Cooke, Julia R. Köhler, Jana Patton-Vogt

**Affiliations:** 1 Department of Biological Sciences, Duquesne University, Pittsburgh, Pennsylvania, USA; 2 Department of Infectious Diseases, Boston Children’s Hospital/Harvard Medical School, Boston, Massachusetts, USA; The University of Texas Health Science Center at Houston, Houston, Texas, USA

**Keywords:** phosphate metabolism, phospholipids, *Candida albicans*, glycerophosphodiesters, phosphate, cell signaling

## Abstract

The fungal pathogen *Candida albicans* must acquire phosphate to colonize, infect, and proliferate in the human host. *C. albicans* has four inorganic phosphate (P_i_) transporters, Pho84 being the major high-affinity transporter; its cells can also use glycerophosphocholine (GPC) as their sole phosphate source. GPC is a lipid metabolite derived from deacylation of the lipid phosphatidylcholine. GPC is found in multiple human tissues, including the renal medulla, where it acts as an osmolyte. *C. albicans* imports GPC into the cell via the Git3 and Git4 transporters. Internalized GPC can be hydrolyzed to release P_i_. To determine if GPC import and subsequent metabolism affect phosphate homeostasis upon P_i_ limitation, we monitored growth and phenotypic outputs in cells provided with either P_i_ or GPC. In *pho84*∆*/*∆ mutant cells that exhibit phenotypes associated with P_i_ limitation, GPC provision rescued sensitivity to osmotic and cell wall stresses. The glycerophosphodiesterase Gde1 was required for phenotypic rescue of osmotic stress by GPC provision. GPC provision, like P_i_ provision, resulted in repression of the PHO regulon and activation of TORC1 signaling. P_i_ uptake was similar to GPC uptake when phosphate availability was low (200 µM). While available at lower concentrations than P_i_ in the human host, GPC is an advantageous P_i_ source for the fungus because it simultaneously serves as a choline source. In summary, we find GPC is capable of substituting for P_i_ in *C. albicans* by many though not all criteria and may contribute to phosphate availability for the fungus in the human host.

**IMPORTANCE:**

*Candida albicans* is the most commonly isolated species from patients suffering from invasive fungal disease. *C. albicans* is most commonly a commensal organism colonizing a variety of niches in the human host. The fungus must compete for resources with the host flora to acquire essential nutrients such as phosphate. Phosphate acquisition and homeostasis have been shown to play a key role in *C. albicans* virulence, with several genes involved in these processes being required for normal virulence and several being upregulated during infection. In addition to inorganic phosphate (P_i_), *C. albicans* can utilize the lipid-derived metabolite glycerophosphocholine (GPC) as a phosphate source. As GPC is available within the human host, we examined the role of GPC in phosphate homeostasis in *C. albicans*. We find that GPC can substitute for P_i_ by many though not all criteria and is likely a relevant physiological phosphate source for *C. albicans*.

## INTRODUCTION

*Candida albicans* is the species most commonly isolated from patients suffering from invasive fungal disease (1). It is also a commensal organism colonizing oral mucosa and the gastrointestinal and genitourinary tracts of many healthy individuals (2, 3). Within these host niches, *C. albicans* competes for resources with the host flora, necessitating a variety of strategies to utilize host nutrients (4, 5). Phosphate is a required nutrient and plays a key role in *C. albicans* survival and growth and in its ability to invade the host. Several genes involved in phosphate acquisition and homeostasis are upregulated during *C*. *albicans* infection (6–8).

**Fig 1:**
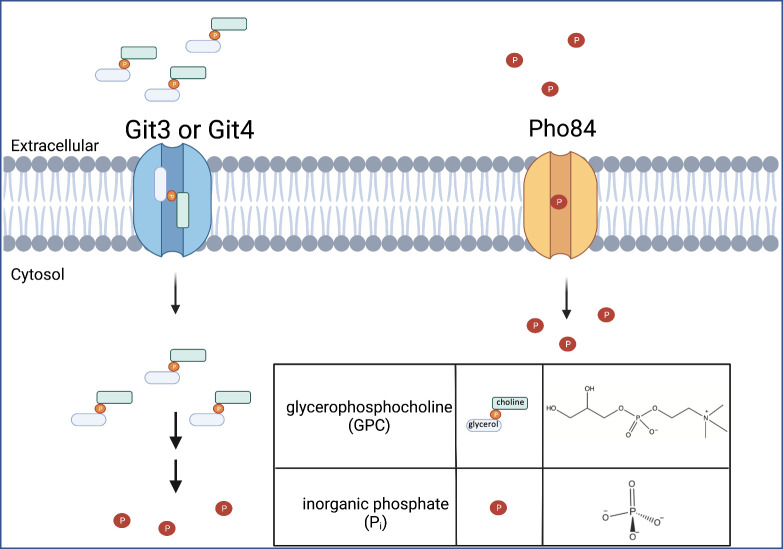
Glycerophosphocholine (GPC) and P_i_ acquisition in *Candida albicans.* Representation of GPC import through Git3 or Git4 followed by its two-step catabolism to release P_i_. P_i_ import through Pho84, a major high-affinity P_i_ transporter in *C. albicans*. Made in Biorender.

The phosphate homeostatic system in *C. albicans*, known as the PHO regulon, is conserved among human fungal pathogens. The major transcriptional regulator of the PHO regulon is Pho4 (9, 10). Cells lacking Pho4 are unable to upregulate many genes involved in phosphate acquisition and are more sensitive to a variety of stressors, including osmotic and cell wall stresses (6, 10). Pho84, a major high-affinity inorganic H+/phosphate (P_i_) symporter, a member of the major facilitator superfamily, is among the targets of Pho4. Pho84 is one of four predicted phosphate importers in *C. albicans* (Fig. 1). Deletion of *pho84* causes increased sensitivity to external stressors, increases ROS levels, and diminishes levels of nucleotide sugars required for cell wall synthesis (11, 12). Loss of Pho84 also causes *in vitro* hyphal growth defects and decreases the virulence of *C. albicans* in a *Drosophila* model and in murine models of oropharyngeal and systemic candidiasis (11).

In addition to P_i_ transporters, Pho4 also regulates the glycerophosphodiester transporters, Git3 and Git4 (13) (Fig. 1). Glycerophosphodiesters are common lipid metabolites produced by cytosolic and secretory phospholipases of the A and B type that deacylate glycerophospholipids such as phosphatidylcholine (PC) (14–16). Importantly, phospholipase activities from both *C. albicans* and the human host contribute to GPC production through hydrolysis of available PC derived from their own or each other’s cellular membranes (14–18). Metabolomic studies have identified GPC in serum, in the gastrointestinal and urinary tract, and in various human tissues (19–21).

The Git transporters are homologs of the *S. cerevisiae* Git1 transporter, which is specific for the glycerophosphodiester, glycerophosphoinositol (GPI) (15), not to be confused with glycosylphosphatidylinositol, the lipid anchor linking proteins to the membrane. Whereas *S. cerevisiae* has a single *GIT1* gene, *C. albicans* has 4 Git1 homologs, Git1–4 (22). While Git2 has yet to be characterized, Git1 is specific for GPI, and Git3 and Git4 transport GPC (Fig. 1). GPC import occurs under phosphate replete conditions and is upregulated by phosphate limitation. Of note, the rate of GPC transport in *C. albicans* under phosphate-limiting conditions is roughly 50× greater than that observed for *S. cerevisiae*, suggesting differential importance for GPC acquisition between the two organisms (13). Indeed, inhibiting GPC import by deleting *git2-4* results in a decrease in the virulence of *C. albicans* in a mouse model of bloodstream infection (13). Once internalized, GPC can be hydrolyzed to choline and glycerol-3-phosphate by glycerophosphodiesterases. A single glycerophosphodiesterase, Gde1, has been characterized, and its expression is also upregulated by phosphate limitation (13). In order to release P_i_ from glycerol 3-phosphate, a phosphomonoesterase is required. Phosphomonoesterases such as Rhr2 and Dog1 have been identified in *C. albicans*; however, there may be other phosphomonoesterases that can hydrolyze glycerol 3-phosphate (23, 24).

Based on limited quantitative studies, GPC appears to be available at concentrations of ≥1 order of magnitude lower than those of P_i_ in serum (19, 25–27). Nonetheless, our previous findings led us to hypothesize that GPC import and subsequent metabolism affect phosphate homeostasis. To test this hypothesis, we examined the ability of GPC provision to affect established aspects of phosphate limitation and signaling in *C. albicans*. We report that provision of GPC rescued several growth defects of a *pho84*∆*/*∆ mutant. In addition, GPC provision, like P_i_ provision, resulted in repression of the PHO regulon though to a lesser degree. Strikingly, GPC activated TORC1 in cells lacking the major P_i_ transporter Pho84. Through radiolabel uptake analysis, we found that P_i_ uptake was roughly 2× as great as GPC uptake under low total phosphate conditions, but similar to GPC when ambient phosphate concentrations were moderate. To illustrate the nutritional utility of GPC transport and subsequent metabolism for the cell, we employed a choline auxotrophic strain to show that provision of GPC can simultaneously act as both sole phosphate and sole choline source. Overall, our studies indicate that GPC can substitute for P_i_ in several though not all measures of phosphate homeostasis and is likely a physiologically relevant phosphate source *in vivo*.

## MATERIALS AND METHODS

### Strains and media

*C. albicans* strains used in this study can be found in Table 1. Strains were grown aerobically at 30°C unless otherwise stated. Turbidity was monitored by measurement of absorbance at 600 nm (A_600_) on a BioMate 150 Thermo Scientific spectrophotometer. The medium used for this study was synthetic complete (SC) (yeast nitrogen base [YNB]) containing 2% glucose and amino acids, as described previously (28). Medium phosphate concentrations were controlled by omitting KH_2_PO_4_ (1 g/L) from the synthetic mix and replacing it with KCL (1 g/L). KH_2_PO_4_ or GPC was added back into media at high (10 mM), medium (1 mM), or low (200 µM) concentrations unless otherwise stated. Strains were maintained on YPD agar (yeast extract 10 g, peptone 20 g, and dextrose).

**TABLE 1:** Strains used

Strain	Genotype	Reference
JKC915, wild type (WT)	*HIS1/his1::tetR-FRT*	(29)
JKC1450, *pho84*∆*/*∆	*pho84::HIS1/pho84::ARG4 his1/his1:: tetR-FRT arg4/arg4 IRO1/iro1*Δ*::*λ*imm434 URA3/ura3*Δ*::*λ*imm434*	(30)
JKC1588, *pho84*∆*/*∆ *+ PHO84*	*PHO84-FRT/pho84::ARG4 LEU2/leu2::C.d. HIS1 his1/his1:: tetR-FRT arg4/arg4 URA3/ura3::λimm434 IRO1/iro1*Δ*::*λ*imm434*	(30)
JKC1659	*HIS1/his1Δ:: tetR-FRT* *PHO84/PHO84 promoter-GFP-NAT1-PHO84*	(12)
*git2,3,4*∆*/*∆ + pDDB78	*ura3*Δ::λ*imm434 arg4*::*hisG his1*::*hisG*::*pHIS1 git2,3,4*::*ARG4* *git2,3,4*::*URA ura3*Δ::λ*imm434 arg4*::*hisG his1*::*hisG 3*	(13)
*gde1*∆*/*∆ + pDDB78	*ura3*Δ::λ*imm434 arg4*::*hisG his1*::*hisG*::*pHIS1 gde1*::*ARG4* *gde1*::*URA ura3*Δ::λ*imm434 arg4*::*hisG his1*::*hisG 3*	(13)
*gde1*∆*/*∆ *+ GDE1*	*ura3*Δ::λ*imm434 arg4*::*hisG his1*::*hisG*::*pHIS1 gde1*::*ARG4* *gde1*::*URA ura3*Δ::λ*imm434 arg4*::*hisG his1*::*hisG 3*	(13)

### Growth assays

Overnight cultures were used to inoculate 200 µL of media at A_600_ = 0.1 in a 96-well plate. Plates were incubated at 30°C, with intermittent shaking prior to each reading using a Molecular Devices SpectraMax i3 instrument. A_600_ readings were taken at 30-minute intervals, and time zero values were subtracted from each timepoint to reflect overall growth. Data points represent the mean and standard deviation of a minimum of three independent replicates.

### Acid releasable inorganic phosphate assay

Acid-labile phosphate was measured by use of a colorimetric molybdate assay as previously described (30, 31). Briefly, overnight cultures were grown overnight in low phosphate YNB containing 200 µM P_i_. Cells were washed twice, reinoculated at A_600_ = 0.1 in YNB containing 200 µM P_i_ or 200 µM GPC, and grown to a mid-log phase. Cells were then harvested and resuspended in 500 µL 0.1% Triton X-100 and lysed using zirconia/silica bead homogenization. Lysate protein concentrations were determined using a BCA Protein Assay Kit (Pierce). One hundred micrograms of whole-cell lysate was then boiled for 30 minutes in 1 M HCl before phosphate quantification using the colorimetric molybdate assay in biological triplicate.

### ^32^P-orthophosphate and ^14^C-choline-glycerophosphocholine uptake assays

Uptake assays were altered from references 32, 33. Cells were pregrown in low phosphate conditions (200 µM KH_2_PO_4_). Cultures were then reinoculated in the indicated media conditions and grown into log phase at 30°C. Once in a log phase, cultures were reinoculated at A_600_ = 0.1 and provided with either ^32^P-orthophosphate or ^14^C-choline-glycerophosphocholine at the indicated concentrations in separate cultures. After 1 hour, 1-mL aliquots were removed from each culture, centrifuged briefly, and separated into extracellular and cellular fractions. Radioactivity in each fraction was measured using liquid scintillation counting.

### *PHO84* promotor induction analysis

Cells of genotype *PHO84/pPHO84-GFP-NAT1-PHO84* were grown in YPD liquid medium with additional 10 mM P_i_ for 16 hours in order to maximally repress the *PHO84* promoter and washed three times with 0.9% NaCl. Cultures were adjusted to A_600_ = 0.01 in synthetic complete with increasing concentrations of KH_2_PO4 or GPC (0.05, 0.1, 0.2, 0.4, and 0.5 mM) and 50 µL/well of eight technical replicates for each condition was inoculated into a black 384-well plate with a transparent bottom. During incubation at 30°C, A_600_ and GFP signal (Ex 485/20 nm; Em 528/20 nm) were recorded every 30 minutes at gain 50 in a Synergy 2 BioTek Plate Reader for 18 hours. Readings were graphed in GraphPad Prism at the 16-hour timepoint.

### P-S6 western blotting in Pho84 strains with GPC

Cell lysis and western blotting were performed as described in (34). Rabbit anti-P-S6 (Cell Signaling Technology, #9611L) was used as the primary antibody. The loading control was Rabbit anti-Cdc28 (PSTAIRE, Santa Cruz #sc-53). Anti-rabbit IgG (Cell Signaling Technology, #7074S) was used as the secondary antibody. For densitometry, ImageJ (imagej.net/welcome) software (opensource) was used to quantitate signals obtained from Azure biosystems c600.

## RESULTS

### Provision of GPC rescued hypersensitivity of *pho84∆/∆* cells to osmotic and cell wall, but not peroxide, stresses

**Fig 2:**
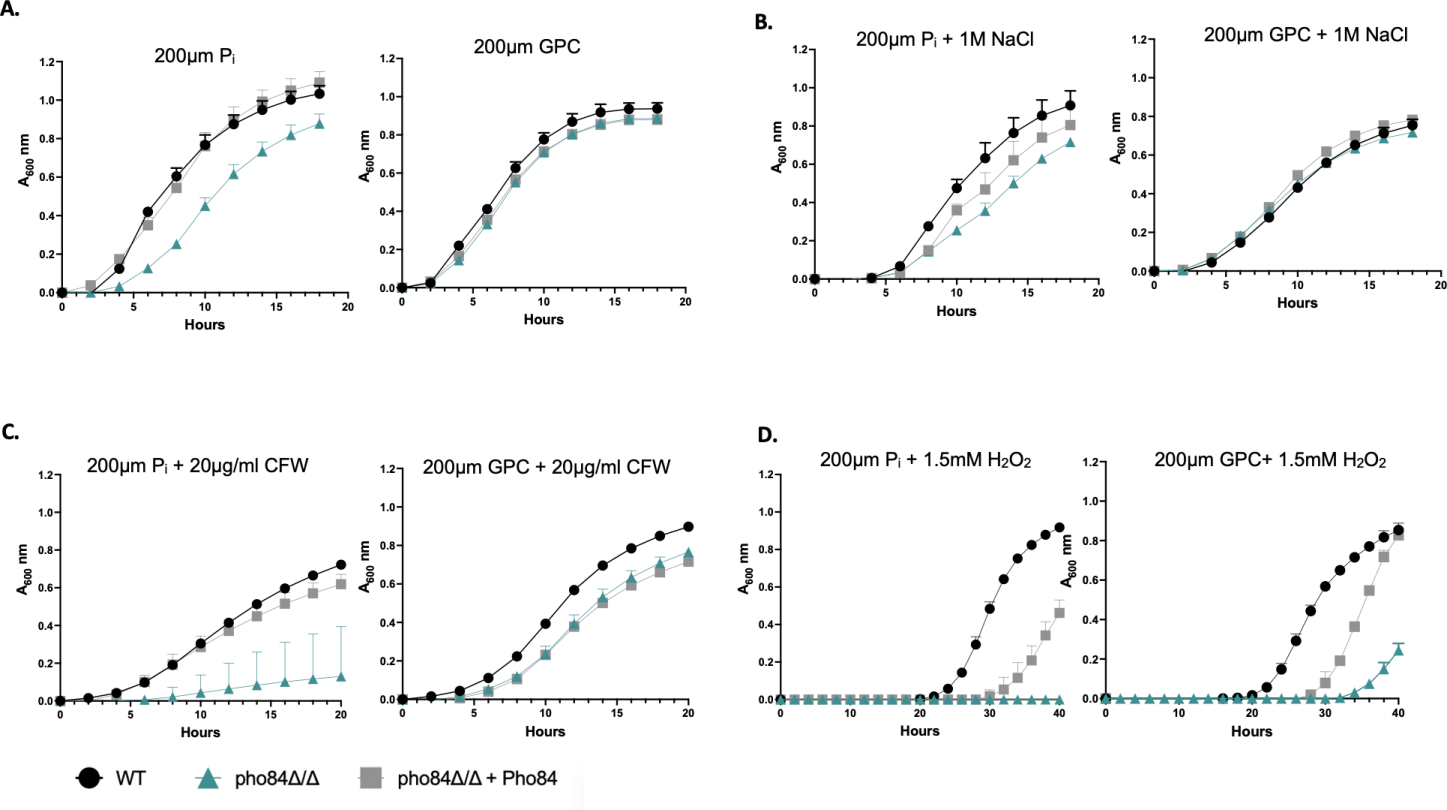
GPC rescued phosphate starvation phenotypes. (**A)** Cells were grown overnight in YNB containing 200 µM P_i_. Cells were then reinoculated into phosphate-free YNB containing either 200 µM P_i_ or 200 µM GPC at an A_600_ of 0.1, monitored every 30 minutes. (B) Grown as in panel A with the addition of 1 M NaCl to induce osmotic stress. (C) Grown as in panel A with the addition of 20 µg/mL calcofluor white to induce cell wall stress. (D) Grown as in panel A with the addition of 1.5 mM H_2_O_2_ to induce oxidative stress. Data are displayed as the mean ± standard deviation of at least three replicates.

Pho84 is the major high-affinity P_i_ transporter in *C. albicans* (30), and its function can only partially be substituted by other phosphate transporters, such as Pho89 and Pho87 (Table 2). In the absence of Pho84, or of its major transcriptional regulator, Pho4, *C. albicans* becomes more susceptible to a variety of stresses including osmotic, cell wall, and oxidative stresses (8–12). We performed growth experiments to see if the provision of GPC can alleviate growth defects and stress hypersensitivities associated with phosphate limitation caused by loss of *PHO84*. As shown in Fig. 2A (top left), a *pho84*∆*/*∆ mutant displayed a growth defect in low phosphate (200 µM P_i_) conditions in comparison to WT (Pho84 is primarily responsible for transport at this concentration, Table 2). Provision of 200 µM GPC instead of P_i_ in the media rescued this phenotype. A *pho84*∆*/*∆ mutant also showed increased sensitivity to osmotic stress caused by 1M NaCl addition when grown in media containing 200 µM P_i_ in comparison to WT (Fig. 2B, top right). When provided 200 µM GPC instead of P_i_, this phenotype was rescued restoring growth to WT levels. Similarly, during cell wall stress induced by exposure to 20 µg/mL of the chitin-binding compound calcofluor white (CFW), *pho84*∆*/*∆ mutant cells grew poorly (Fig. 2C, bottom left), as we showed previously (12). This phenotype was rescued by provision of 200 µM GPC as phosphate source, which restored growth similar to the WT in these conditions (Fig. 2C, bottom left). Hypersensitivity of *pho84*∆*/*∆ cells to peroxide stress (11) (Fig. 2D, bottom right) induced by 1.5 mM H_2_O_2_ was not clearly rescued by provision of GPC, although some growth began to occur in *pho84*∆*/*∆ cells after 30 hours. Of note, both WT and reintegrant strains displayed long lag times (note *x*-axis) upon provision of either P_i_ or GPC in this experiment, indicating that 1.5 mM H_2_O_2_ presented the cells with a severe stress. One interpretation is that the two-step requirement to release free P_i_ from GPC precludes efficient rescue at this H_2_O_2_ concentration. Further experimentation will be required to test whether *pho84*∆*/*∆ cells’ elevated reactive oxygen species’ content even in the absence of exogenous oxidative stress, and their hypersensitivity to superoxide and peroxide stresses (11), can be rescued by provision of GPC.

**TABLE 2:** Comparison of P_i_ and GPC import^*a*^

Strain	Concentration (P_i_ + GPC)	*GPC uptake (per ODU)	*P_i_ uptake (per ODU)
WT	100 µM + 100 µM	7.43 ± 0.66 nmol	10.6 ± 1.27 nmol
*git2-4*∆*/*∆*, PHO84+/+*		0.00 ± 0.72 nmol	ND
*pho84*∆*/*∆*, GIT2-4+/+*		ND	0.00 ± 0.45 nmol
WT	250 µM + 250 µM	11.6 ± 1.26 nmol	23.25 ± 3.31 nmol
*git2-4*∆*/*∆*, PHO84+/+*		0.00 ± 0.65 nmol	ND
*pho84*∆*/*∆*, GIT2-4+/+*		ND	0.00 ± .07 nmol

^*a*^ Cells were grown to log phase in YNB containing equivalent amounts of P_i_ and GPC at two concentrations: very low phosphate (100 µM P_i_ + 100 µM GPC) or low phosphate (250 µM P_i_ + 250 µM GPC) conditions. Cells were then reinoculated into the same nutrient conditions at an A_600_ of 0.1, but in media in which only one of the compounds was radiolabeled (^14^C-choline-GPC or ^32^P-orthophosphate, indicated by an asterisk at the top of the data column). Samples were incubated for 1 hour at which point cells were harvested and cellular radioactivity was determined per 1 ODU. Data represent the mean and standard deviation of biological triplicates. ND, not done.

Overall, these results suggest that GPC is transported into the cell and enters cellular metabolism efficiently enough to counter major phenotypes associated with severe P_i_ limitation as experienced by cells lacking *PHO84* in low ambient phosphate.

### Gde1 was required for GPC utilization as a phosphate source

**Fig 3:**
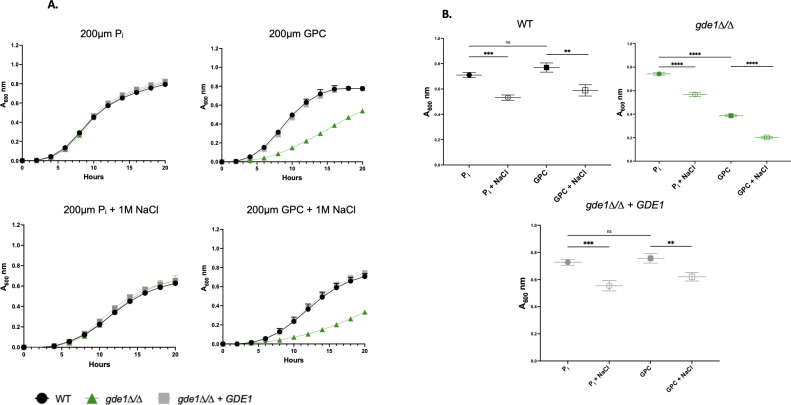
A *gde1*∆*/*∆ mutant displayed defective growth on GPC. (**A)** Cells were grown overnight in YNB containing 200 µM P_i_. Cells were then reinoculated into phosphate-free YNB containing either 200 µM P_i_ or 200 µM GPC at an A_600_ of 0.1 with or without 1 M NaCl. A_600_ was monitored every 30 minutes using a plate reader. Data are displayed as the mean and standard deviation of at least three replicates. (B) The 16-hour timepoint from the data in A. A one-way ANOVA was performed. **, *P* < 0.001; ***, *P* < 0.0005; ****, *P* < 0.0001.

Uptake of GPC requires the Git3 and Git4 transporters (13). Once internalized, GPC is hydrolyzed by the glycerophosphodiesterase Gde1 into choline and glycerol-3 phosphate (13). To verify that GPC must be hydrolyzed prior to its utilization as a phosphate source under stressed and non-stressed conditions, we employed a *gde1*∆*/*∆ mutant subjected to NaCl stress. As shown in Fig. 3A, WT and *gde1*∆*/*∆ grew identically when P_i_ was the phosphate source, both in the presence and in the absence of 1 M NaCl. However, when GPC was provided as the phosphate source, *gde1*∆*/*∆ showed a growth defect in the absence of stress, and that defect increased in the presence of 1 M NaCl. The growth defect of *gde1*∆*/*∆ is shown in Fig. 3B, where only the 16-hour timepoint is presented. Of note, loss of Gde1 does not completely inhibit growth on GPC, indicating that there are other, as yet uncharacterized, enzymes that can hydrolyze GPC (13).

### GPC and P_i_ provision supported similar levels of intracellular acid-labile phosphate

To directly determine how provision of GPC compares with provision of P_i_ in terms of internal phosphate stores, we quantified acid-labile phosphate within cells. Boiling acid treatment releases phosphate from polyphosphates and has been used to determine estimates of free P_i_ arising from polyphosphates in yeast (30, 35).

**Fig 4:**
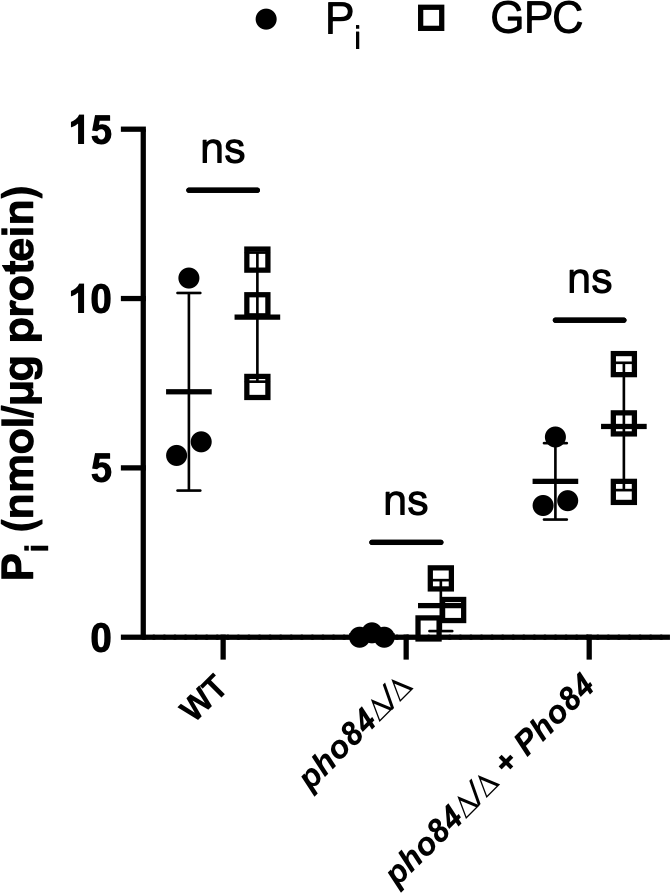
Internal phosphate levels were similar when cells were provided with either GPC or P_i_. Cells were grown overnight in YNB containing 200 µM P_i_. Cells were then reinoculated into phosphate-free YNB containing either 200 µM P_i_ or 200 µM GPC at an A_600_ of 0.2 and grown to mid-log phase. Cells were then harvested and assayed for acid-labile phosphate. Data represent the mean and standard deviation of biological triplicates. A one-way ANOVA was performed.

As shown in Fig. 4, GPC and P_i_ provision supported similar levels of total free and acid-labile internal phosphate in a wild-type strain. This implies that despite the two steps of catabolism needed to release free phosphate from GPC, internal phosphate stores are not negatively impacted. Previous studies have shown that in the absence of Pho84, there is a significant decrease in the amount of acid-labile phosphate, interpreted to be representative of polyphosphate storage (30, 35). We have repeated those findings and report that provision with GPC was not able to rescue this phenotype (Fig. 4). Given the predicted lower P_i_ content of *pho84*∆*/*∆ cells at the beginning of the experiment (30), the provided GPC may have only been sufficient to enable biomass addition but not replenishment of polyphosphate stores in these cells.

### GPC repressed *PHO84* promoter activation

The PHO regulon is the key homeostatic mechanism for phosphate import and intracellular phosphate distribution. Pho4 is the primary transcriptional activator of the PHO regulon, upregulating the expression of *PHO84*, the genes encoding the GPC importers Git3 and Git4, and the glycerophosphodiesterase Gde1 among many other genes (9, 10). Pho4 activity increases as the P_i_ available within the cell decreases. To assess how provision of GPC impacts PHO regulon signaling, we measured the fluorescence produced through activation of the *PHO84* promoter driving GFP in a strain containing two functional *PHO84* alleles (12) as a proxy for Pho4 transcriptional activity.

**Fig 5:**
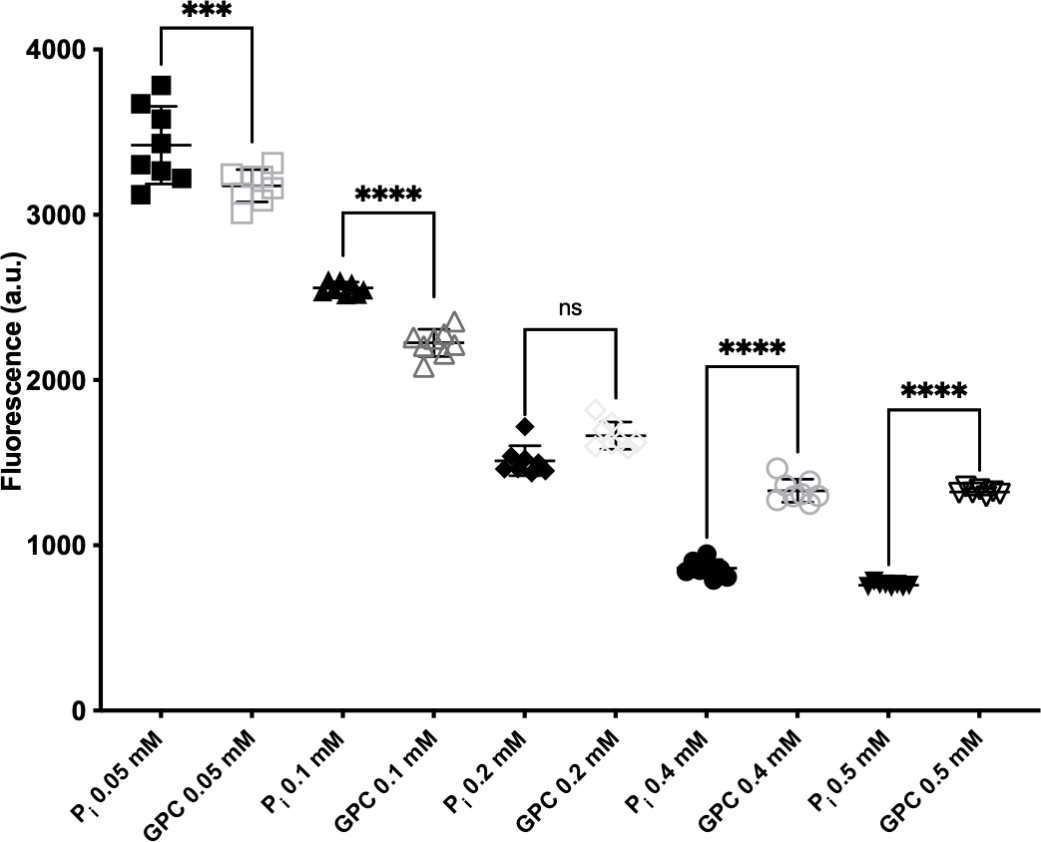
GPC repressed the *PHO84* promoter. Cells expressing *GFP* under the control of the *PHO84* promoter (JKC1659) were pregrown in YPD with an additional 10 mM P_i_ overnight. Cells were then inoculated at an OD_600_ = 0.01 into media without P_i_ with indicated P_i_ or GPC concentrations. The fluorescent signal and A_600_ were followed over 18 hours. The 16-hour timepoint is shown. Data represent the mean and standard deviation of eight biological replicates. A one-way ANOVA was performed. a.u., arbitrary unit ***, *P* < 0.0005; ****, *P* < 0.0001.

As expected and previously shown (12), fluorescence from p*PHO84*-GFP decreased as P_i_ concentration in the medium was increased from 0.05 mM to 0.5 mM (Fig. 5). When GPC was provided as the phosphate source, a comparable decrease in p*PHO84*-GFP fluorescence occurred (Fig. 5). Thus, GPC repressed the PHO regulon in a qualitatively similar manner to P_i_. At 0.05 mM and 0.1 mM P_i_, we observed a statistically significant but small increase in repression (roughly 110%) of p*PHO84*-GFP by GPC as compared with P_i_, a result for which we have no obvious explanation. At 0.2 mM, repression by both phosphorus sources appeared equivalent. A larger quantitative difference was noted at the 0.4 mM and 0.5 mM levels, where P_i_ repressed expression to a greater extent (roughly 150%) than GPC. Pho4 also regulates the expression of Gde1, the enzyme needed for the first step in hydrolysis to release P_i_ by GPC hydrolysis. It may be that at high GPC concentrations, less P_i_ is released from GPC due to insufficient Gde1 production, leading to decreased repression of the PHO regulon.

### GPC activates TORC1 signaling independently of Pho84

The mechanisms by which the cell senses cellular phosphate is an active area of research.

**Fig 6:**
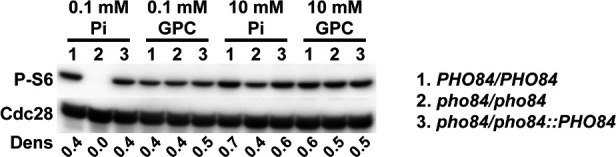
GPC activated TORC signaling. Cells pregrown in YPD overnight were washed three times in 0.9% NaCl and then inoculated into synthetic complete medium with 0.1 mM or 10 mM inorganic phosphate or glycerophosphocholine, at an A_600_ of 0.2. Cells were collected after 2 hours of incubation (200 rpm) at 30°C. Total protein extracts were probed with antibody to phosphorylated Rps6 (P-S6) and Cdc28 as loading control. Dens, signal intensity ratio of P-S6 to Cdc28. (1, PHO84/PHO84, JKC915; 2, pho84/pho84, JKC1450; 3, pho84/pho84::PHO84, JKC1588.) Representative of three biological replicates.

However, it has been established that phosphate, in addition to nitrogen and carbon, is one of the nutrients sensed by the *C. albicans* and *S. cerevisiae* TOR (target of rapamycin) complex 1 (TORC1) signaling pathway (12, 30). TORC1 signaling is highly conserved within eukaryotes and controls cellular growth and proliferation in dependence on nutrient availability. During phosphate starvation or upon *PHO84* deletion, TORC1 signaling is decreased. TORC1 signaling is activated by the upstream GTPase, Gtr1 (12, 30). In a recent study, Pho84 is hypothesized to have transceptor activity, affecting TORC1 signaling in combination with P_i_ import (36). To examine if the provision of phosphorus as GPC is able to activate TORC1 similarly to the provision of P_i_, we monitored the phosphorylation of ribosomal protein S6, a known downstream target of TORC1 (34). In a wild-type strain, provision of GPC activated TORC1 signaling similarly to P_i_ provision. Consistent with previous studies, we found that TORC1 signaling was decreased in *pho84*∆*/*∆ compared with wild-type cells when they were provided with P_i_ (Fig. 6). When GPC was provided to *pho84*∆*/*∆ cells at concentrations of 0.1 and 10 mM, TORC1 signaling was restored. Thus, GPC activated TORC1 independently of Pho84 to return signaling to wild-type levels (Fig. 6).

### Comparison of GPC and P_i_ transport

In the experiments represented in Fig. 2–6, either GPC or P_i_ was provided as the sole phosphate source. In the human host, GPC and P_i_ are both available albeit at different concentrations and compartment distributions (37). Human serum P_i_ is measured routinely as part of standard electrolyte panels and ranges from 0.8 mM to 1.3 mM (26, 27). GPC concentrations in serum have been measured much less frequently and range from 3 mM to 35 mM (19, 25). However, the relative concentrations of GPC and P_i_ in host microenvironments have not been established. Further, increased GPC transport capacity of *C. albicans* compared with *S. cerevisiae*, regulation of its transporters Git3 and Git4 by the PHO regulon, and their contribution to *C. albicans* virulence argue for a significant role of GPC in the fungus’ nutritional repertoire in the host.

We therefore examined the transport of GPC and P_i_ when both metabolites were available. Equimolar amounts of P_i_ and GPC were provided in experiments in which one of the two was radiolabeled (^14^C-choline-GPC or ^32^P-orthophosphate). We used low and moderate total phosphate concentrations, either 200 µM total phosphate (100 µM P_i_ plus 100 µM GPC) or 500 µM total phosphate (250 µM P_i_ plus 250 µM GPC). In this way, we compared two conditions in which the PHO regulon was induced, and therefore, the transporters of both GPC and P_i_ were expected to be expressed, albeit at higher levels in lower phosphate concentrations. To control for a background level of GPC transport, we used a *git2-4*∆*/*∆ strain that showed negligible GPC transport under the conditions examined. We also observed negligible P_i_ transport in *pho84*∆*/*∆ cells (Table 2). At a total ambient phosphate concentration of 200 µM, P_i_ and GPC were imported at similar rates in the course of the 1-hour assay: roughly 10 nanomoles of P_i_ as compared with 7 nanomoles of GPC per mL of cell suspension at an OD_600_ of 1 (ODU) (Table 2). At 500 µM ambient phosphate, P_i_ import was roughly 2× greater than that of GPC: 23 nanomoles of P_i_ as compared with 11 nanomoles of GPC (Table 2). We concluded that when both sources of phosphate were available at the same concentration, GPC was imported at a substantial rate, especially when P_i_ was limiting. GPC transport may be more quickly saturable since P_i_ import increased more with increasing ambient P_i_ availability than GPC import.

### GPC was able to act as both a phosphate and a choline source

**Fig 7:**
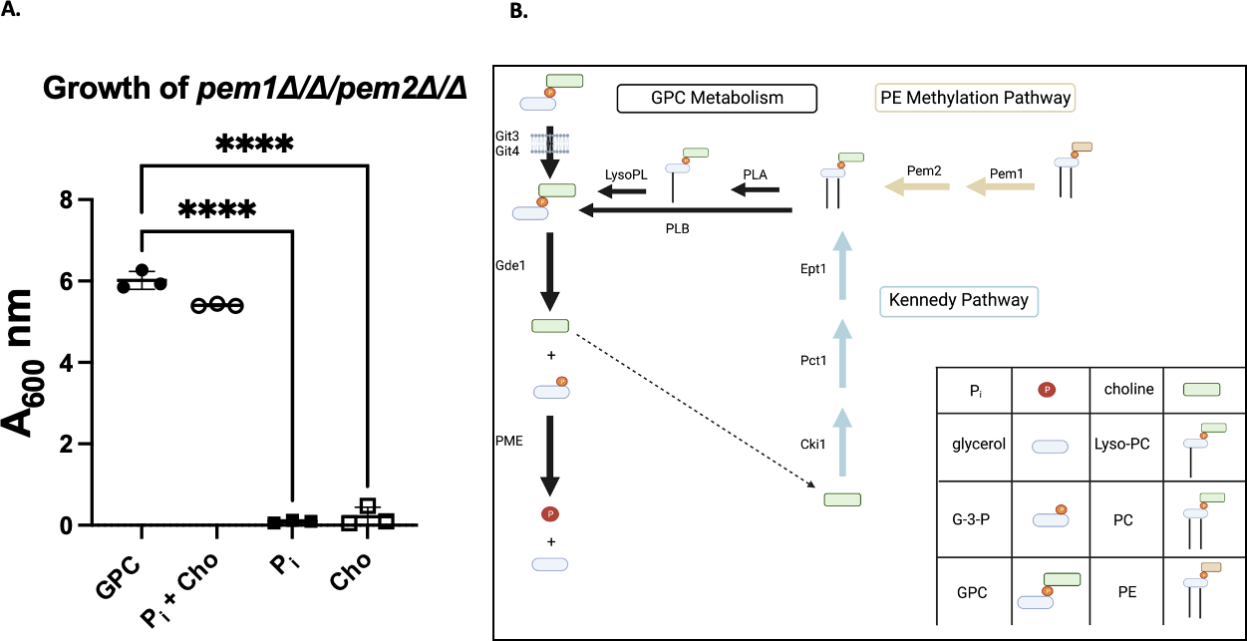
GPC served simultaneously as a P_i_ and choline source. (**A)** *pem1*∆*/*∆*pem2*∆*/*∆ strain, a choline auxotroph, was grown overnight in YNB supplemented with 200 µM choline. Cells were then starved of phosphate and choline for 8 hours before restarting in phosphate-free YNB supplemented with either 200 µM GPC, 200 µM P_i_, 200 µM choline (cho), or 200 µM P_i_ plus 200 µM choline as indicated. Cells were started at an A_600_ of 0.2 and allowed to grow for 24 hours. Data represent the mean and standard deviation of biological triplicates. (B) Schematic of GPC and PC metabolism. GPC production and catabolism is indicated by black arrows. The Kennedy pathway for PC biosynthesis is shown in blue, and the PE methylation pathway for PC biosynthesis is shown in yellow. Shown in black is GPC production through the deacylation of PC by phospholipase of the b type or through a single deacylation of PC by a phospholipase of the A type to produce Lyso-PC followed by another deacylation by a lysophospholipase. GPC, glycerophosphocholine; G-3-P, glycerol-3-phosphate; P_i_, inorganic phosphate; LPC, lyso-phosphatidylcholine; PC, phosphatidylcholine; PE, phosphatidylethanolamine; PLB, phospholipase B; PLA, phospholipase A; LysoPl, lysophospholipase; PME, phosphomonoesterase . A one-way ANOVA was performed. ****, *P* < 0.0001. Made in Biorender.

We used a choline auxotrophic strain to test the possibility that GPC can simultaneously act as the sole phosphate and the sole choline source (Fig. 7A). The *pem1*∆*/*∆ *pem2*∆*/*∆ strain is a choline auxotroph because it lacks the phosphatidylethanolamine methylation pathway, leaving the Kennedy pathway as the sole biosynthetic pathway for phosphatidylcholine biosynthesis in *C. albicans* (38). When this strain was grown without exogenous P_i_ or choline, provision of GPC was able to support growth similarly to media containing both P_i_ and choline (Fig. 7B). We concluded that GPC hydrolysis sufficed to simultaneously provide choline and phosphate to *C. albicans*.

## DISCUSSION

GPC is a ubiquitous lipid metabolite produced by phospholipases in bacteria (39), fungi (15, 40), and mammalian cells (17). GPC is found in human tissues and fluids including serum, prostate, breast tissue, renal cells, saliva, blood, breast milk, and cerebrospinal fluid (17, 19, 41–43). Based on data contained in the human metabolome database (https://hmdb.ca), GPC levels that have been reported thus far have ranged roughly from 1 µM in saliva to 3–35 µM in serum to 30–500 µM in breast milk (19, 25, 41, 44, 45), with the concentrations varying widely depending upon the study and the methodology employed. Although quantitative metabolomic data on GPC are limited, it is clear that serum P_i_ (at roughly 1 mM [26, 27]) is more abundant than serum GPC (19, 25). However, the relative concentrations of GPC and P_i_ in host microenvironments have not been established and are undoubtedly dynamic, as GPC is liberated through phospholipase-mediated PC hydrolysis, during which it may reach higher concentrations than those found in serum at a steady state, while serum and cytosolic P_i_ concentrations are highly regulated (17, 46, 47). Both phospholipase B1 and B5 are required for full virulence of *C. albicans*. One potential mechanism for these phospholipases in pathogenesis is the release of GPC from host cells’ plasma membranes that provides phosphorus as well as choline for the fungus (14, 48–51). GPC is one of four major renal osmolytes found in tubular cells of the renal medulla during mammalian dehydration states, along with glycine betaine, myo-inositol, and sorbitol (52). In renal cells, GPC has been quantified by mass instead of volume and has been reported to be roughly 18 nmol/mg protein (53, 54). In some human candidiasis syndromes and in the murine intravenous infection model, the kidney is a major target for disseminated candidiasis (55), though typically, the renal cortex and medulla are equally infected. Further work, especially regarding the contribution of fungal phospholipases to GPC release, should shed more light on these questions.

*C. albicans* upregulates high-affinity phosphate transporters during invasive infection suggesting that the host environment mimics P_i_ starvation conditions (56–62). At the human serum P_i_ concentration of 0.8–1.3 mM (26, 27), *C. albicans* would be expected to experience P_i_ sufficiency, but given the acidic optima of all but one of its P_i_ importers (our unpublished data), their activity may be inefficient at the normal pH of human serum (pH 7.35–7.45). An analogous phenomenon was observed in the opportunistic fungal pathogen *Cryptococcus neoformans* and called “alkaline pH-simulated nutrient deprivation” (63, 64). We examined the possibility that GPC is among the sources of phosphorus used by *C. albicans* in the host.

Previous studies have established that *C. albicans* can utilize GPC efficiently as a phosphate source (13). Importantly, loss of the Git3 transporter results in decreased virulence in a mouse model of bloodstream infection (13). We demonstrate here that provision of equimolar GPC can rescue several growth phenotypes associated with phosphate limitation imposed by the lack of *PHO84* (Fig. 2). Further, provision of either P_i_ or GPC at equimolar concentrations results in similar levels of total intracellular phosphate in a WT strain, despite the need for a two-step catabolic process to release P_i_ from GPC. Provision of GPC did not rescue P_i_ stores of *pho84*∆*/*∆ cells (Fig. 4). One interpretation could be that the amount of GPC provided was not enough to support growth of *pho84*∆*/*∆ cells which requires incorporation of phosphorus into many macromolecules and to simultaneously restore depleted internal phosphate stores over the time span of the assay.

To test whether GPC is used in conditions where both P_i_ and GPC are available at equimolar concentrations, we examined the import of each phosphorus source in the presence of the other. GPC import is similar to P_i_ under low ambient phosphate conditions and measures roughly half as much as P_i_ import in moderate ambient phosphate (Table 2). Both import and hydrolysis of GPC are regulated by the PHO regulon; therefore, P_i_ is the most readily usable form of phosphorus (8, 13). In moderate phosphate conditions, P_i_ appears to be preferentially selected over GPC and the molecular mechanism of this selectivity remains to be discovered. Future work will examine whether the GPC transporters, Git3/4, are saturable at lower transport rates than Pho84, whether their expression is downregulated earlier than that of Pho84 in rising ambient P_i_ concentrations, and whether mechanisms beyond the PHO regulon determine their expression and activity. GPC transporters Git3/4 compensate for loss of *PHO84* under conditions where the other P_i_ transporters which are present in the *pho84*∆*/*∆ mutant cannot (Fig. 2 and 3), so that 200 µM GPC rescues phenotypes in a *pho84*∆*/*∆ mutant that cannot be rescued by 200 µM P_i_.

GPC has the potential to provide the cell with choline and glycerol in addition to phosphate. Strains lacking a functional PE methylation pathway are choline auxotrophs as they require choline to make PC via the CDP-choline pathway. It has been shown previously that GPC can act as a choline source (38). Here, we further demonstrate the robustness of GPC import and catabolism by showing that GPC can simultaneously act as both a choline and phosphate source in a *pem1*∆*/*∆ *pem2*∆*/*∆ mutant (Fig. 7). This variety of metabolic uses for GPC may be one of the reasons that the loss of the major GPC transporter, Git3, leads to decrease in virulence in a mouse model (13).

Induction of the *PHO84* promoter is a readout of PHO regulon activity in *C. albicans* (12) as in *S. cerevisiae* (65). We observed repression of the *PHO84* promoter with increasing provision of both P_i_ and GPC in the medium, though P_i_ was the more potent repressor at equimolar concentrations. Why P_i_ has a stronger effect on the PHO regulon than GPC, when equal amounts of phosphate are delivered intracellularly, e.g., whether Pho84 has transceptor activity toward the PHO regulon, remains to be determined.

The ability of GPC to activate TORC1 signaling in cells lacking *PHO84* (Fig. 6) suggests that it is intracellular P_i_, and not simply a direct signal from Pho84, that provides a crucial stimulus to TORC1. While Pho84 may also have a transceptor activity in addition to its role providing P_i_ to the cytoplasm (30, 36), our current findings indicate that in the presence of sufficient intracellular P_i_ provided by GPC, this activity is not required for TORC1 activation. How intracellular P_i_ availability is signaled to TORC1 remains an area of active investigation.

An open area of inquiry is the complete identification of gene products involved in GPC catabolism. As shown in Fig. 3, growth on GPC as a phosphate source is delayed in a *gde1*∆*/*∆ mutant, but there are undoubtedly other glycerophophodiesterases involved as well. Secondly, the phosphomonoesterase(s) responsible for the release of free phosphate from glycerol-3 phosphate have yet to be identified. While phosphomonoesterases, like Rhr2, Dog1, and multiple others are known, their role in GPC catabolism has not been established, and there are likely other phosphomonoesterase-encoding genes in the *C. albicans* genome (7, 8, 23, 24).

Several aspects of the phosphate deprivation response are conserved among pathogenic and nonpathogenic fungi, including the Pho4 transcriptional regulator (10, 66, 67). However, others have noted that pathogenic fungi have an expanded range of Pho4 targets that include lipid metabolism (63). Lipid metabolism, both synthesis and turnover, is an ongoing process in pathogenic fungi and the human host. Our results show that *C. albicans* has adapted to use GPC, the product of PC metabolism, as part of its phosphate deprivation response, and that the choline released in the process can feed into PC biosynthesis. *C. albicans* GPC importers Git3/4 hence have significant roles in both phosphate homeostasis and lipid biosynthesis. Ongoing studies are exploring the possibility that GPC can be converted to PC through direct acylation, as recently shown in *S. cerevisiae*, plants, and mitis group streptococci (28, 68, 69). Interconvertibility of membrane organic phosphates with free cellular P_i_ via GPC may contribute to *C. albicans* adaptation to insufficient P_i_ access in the host.
